# Predicting diagnostic gene expression profiles associated with immune infiltration in patients with lupus nephritis

**DOI:** 10.3389/fimmu.2022.839197

**Published:** 2022-12-02

**Authors:** Lin Wang, Zhihua Yang, Hangxing Yu, Wei Lin, Ruoxi Wu, Hongtao Yang, Kang Yang

**Affiliations:** ^1^ Nephrology Department, First Teaching Hospital of Tianjin University of Traditional Chinese Medicine, Tianjin University of Traditional Chinese Medicine, Tianjin, China; ^2^ Graduate School, Tianjin University of Traditional Chinese Medicine, Tianjin, China; ^3^ Institute of Traditional Chinese Medicine, Tianjin University of Traditional Chinese Medicine, Tianjin, China; ^4^ Nephrology Department, The First Affiliated Hospital of Henan University of Chinese Medicine, Henan, China

**Keywords:** lupus nephritis, immune infiltration, comprehensive bioinformatics, biomarkers, CIBERSORT

## Abstract

**Objective:**

To identify potential diagnostic markers of lupus nephritis (LN) based on bioinformatics and machine learning and to explore the significance of immune cell infiltration in this pathology.

**Methods:**

Seven LN gene expression datasets were downloaded from the GEO database, and the larger sample size was used as the training group to obtain differential genes (DEGs) between LN and healthy controls, and to perform gene function, disease ontology (DO), and gene set enrichment analyses (GSEA). Two machine learning algorithms, least absolute shrinkage and selection operator (LASSO) and support vector machine-recursive feature elimination (SVM-RFE), were applied to identify candidate biomarkers. The diagnostic value of LN diagnostic gene biomarkers was further evaluated in the area under the ROC curve observed in the validation dataset. CIBERSORT was used to analyze 22 immune cell fractions from LN patients and to analyze their correlation with diagnostic markers.

**Results:**

Thirty and twenty-one DEGs were screened in kidney tissue and peripheral blood, respectively. Both of which covered macrophages and interferons. The disease enrichment analysis of DEGs in kidney tissues showed that they were mainly involved in immune and renal diseases, and in peripheral blood it was mainly enriched in cardiovascular system, bone marrow, and oral cavity. The machine learning algorithm combined with external dataset validation revealed that C1QA(AUC = 0.741), C1QB(AUC = 0.758), MX1(AUC = 0.865), RORC(AUC = 0.911), CD177(AUC = 0.855), DEFA4(AUC= 0.843)and HERC5(AUC = 0.880) had high diagnostic value and could be used as diagnostic biomarkers of LN. Compared to controls, pathways such as cell adhesion molecule cam, and systemic lupus erythematosus were activated in kidney tissues; cell cycle, cytoplasmic DNA sensing pathways, NOD-like receptor signaling pathways, proteasome, and RIG-1-like receptors were activated in peripheral blood. Immune cell infiltration analysis showed that diagnostic markers in kidney tissue were associated with T cells CD8 and Dendritic cells resting, and in blood were associated with T cells CD4 memory resting, suggesting that CD4 T cells, CD8 T cells and dendritic cells are closely related to the development and progression of LN.

**Conclusion:**

C1QA, C1QB, MX1, RORC, CD177, DEFA4 and HERC5 could be used as new candidate molecular markers for LN. It may provide new insights into the diagnosis and molecular treatment of LN in the future.

## 1 Introduction

Lupus nephritis (LN) is one of the most common and serious complications of systemic lupus erythematosus (SLE) (1), with a high morbidity and mortality rate. According to statistics, the annual incidence of SLE worldwide is 1/100,000 to 8.7/100,000, and 40% to 60% of SLE patients have LN at onset (1). About one-third of patients with severe LN develop end-stage renal disease (ESRD) after 10 years. The mortality rate is increased 8-fold compared to the general population (2, 3). Given LN’s high morbidity and mortality, early diagnosis and intervention can significantly reduce CKD and ESRD (4). Kidney biopsy remains the gold standard for diagnosing LN but is somewhat invasive. It is also considered that SLE is a systemic autoimmune disease with multi-organ involvement and multiple autoantibody positivity as the primary clinical features, while peripheral blood is prevalent throughout the body and can reflect systemic activity (5, 6). In addition, peripheral blood contains a variety of LN-associated immune cells, easy accessibility, low cost of the examination, and accurate results, making gene expression analysis of peripheral blood cells an ideal source of LN biomarkers (7). For treatment, LN is usually treated with immunosuppressive therapy such as mycophenolate mofetil or cyclophosphamide and glucocorticoids. On the one hand, they are not specific drugs for the disease and are closely associated with a wide range of adverse effects (8). On the other hand, difficulty adhering to treatment due to potential adverse effects may lead to treatment failure and progression to refractory LN (9, 10). Therefore, it is imperative to develop new candidate biomarkers and find potential therapeutic targets to improve the prognosis of LN patients.

With the development of gene microarray technology and high-throughput technology, the use of bioinformatics methods to mine gene microarray data can quickly and effectively screen differential genes(DEGs). In recent years, it has been widely used in the elucidation of the pathogenic mechanism of diseases and screening of drug therapeutic targets (11–13). Both SLE and LN are largely influenced by genes (14). Therefore, in this study, we used bioinformatics methods to obtain RNA gene matrices from peripheral blood and kidney tissues of LN, respectively, from the GEO database, and performed DEGs enrichment analysis between LN and healthy samples. Two machine learning algorithms were used to screen biomarkers associated with LN, and candidate genes closely related to immune infiltration were further validated in another validation cohort. In this study, CIBERSORT was used for the first time to quantify the proportion of immune cells in LN and normal tissue samples based on gene expression profiles. In addition, we explored the relationship between the identified biomarkers and infiltrating immune cells to provide new ideas for the further prevention and treatment of LNs.

## 2 Materials and methods

### 2.1 Microarray data

In the Gene Expression Omnibus (GEO) database (https://www.ncbi.nlm.nih.gov/geo/), use “Lupus nephritis” as the search term, limit entry type to series, and detection method/data type to expression profiling by array, the organization source is homo sapiens, and all gene expression data related to LNs were retrieved as of October 3rd, 2022. The data sets were differentiated for tissue origin: peripheral blood and kidney tissue. Three eligible gene expression datasets were screened in the renal group: GSE32591 (64 LN, 29 healthy controls), GSE113342 (56 LN, 16 healthy controls), and GSE200306 (79 LN, 19 healthy controls), enrolling a total of 199 patients with LN and 64 healthy controls. Three eligible gene expression data sets were screened in the peripheral blood group: (GSE72798 (30 LN, 17 healthy controls), GSE99967 (29 LN, 17 controls), and GSE81622 (14 LN, 24 controls)’s, which included a total of 73 LN patients and 58 healthy controls.

### 2.2 Data processing and DEG screening

Firstly, convert the probe matrix into a gene matrix according to the probe annotation file. For multiple probes corresponding to the same gene, the average value of the probes is calculated as the final expression value of the gene. Secondly, the two data sets with larger sample size were pooled and used as training groups, GSE99967, GSE72798 and GSE32591, GSE113342 for peripheral blood and kidney tissues respectively, and GSE81622 and GSE200306 for the validation group. The sva package was used to process the batch effect, the limma package was used to analyze theDEGs in the matrix, and the DEGs were selected with the gene expression | log2 fold change, logFC | > 1 and the adjusted P value <0.05 as the screening conditions. Finally, ggplot was used to visualize the DEGs and plot the corresponding volcanoe map.

### 2.3 Functional enrichment analysis of DEGs

The “clusterProfiler” and “DOSE” packages of the R package were used to perform disease ontology (DO) enrichment analysis of DEGs. And gene set enrichment analysis (GSEA) was also used to identify significant pathways between the LN and control groups. The “c2.cp.kegg.v7.4.symbols.gmt” from the Molecular Signatures Database (MSigDB) was used as the reference gene set. If p. adjust < 0.05, the pathways were considered significantly enriched.

### 2.4 Screening of diagnostic biomarkers

Machine learning is a novel algorithm analysis tool, and in this study, the least absolute shrinkage and selection operator (LASSO) combined with support vector machine-recursive feature elimination (SVM-RFE) was applied to the screening of LN biomarkers to predict the status of the disease. LASSO is a regularized regression algorithm using the “glmnet” package in R (15). SVM-RFE is a supervised machine learning technique that can rank features based on recursion to avoid overfitting (16, 17). The genes screened by the two algorithms are intersected and the overlapping genes are the candidate bio-diagnostic markers.

### 2.5 Diagnostic value of biodiagnostic markers in LN

In order to test the accuracy of the biodiagnostic markers screened by machine learning, we generated ROC curves using the “diff gene xp” between the LN group and the normal sample group. The greater the area under curve (AUC), the higher the accuracy of the gene as a diagnostic marker in LN. In the same method, its effectiveness was further verified in the validation group GSE81622/GSE200306.

### 2.6 Identification of immune cell subsets

The CIBERSORT algorithm is a reliable machine learning method based on linear support vector regression (SVR), which is widely used to assess the relative content and dynamic regulatory process of 22 immune cells and is superior to other methods for identifying human immune cell phenotypes in terms of noise and unknown mixed content (18, 19). In order to understand the relative proportions of different immune cells in the LN sample, this study used the R language program and linked CIBERSORT to simulate and calculate the transcription feature matrix of 22 immune cells, and the number of calculations was set to 1,000. Based on the P value<0.05, use the “corrplot” package to draw correlation heat maps to visualize the correlation between 22 infiltrating immune cells; use “vioplot” to draw a violin chart to visualize the differences between the immune cells of the LN group and the control group.

### 2.7 Correlation analysis between bioiagnostic markers and infiltrating immune cells

Correlation analysis between genes and immune cells was performed using Spearman correlation coefficient. For those with p < 0.05, the results were visualized using the “ggplot2” package.

### 2.8 Correlation of bioiagnostic markers with clinicopathological features

To explore the correlation of candidate gene markers with different pathological types, relapse frequency, renal tubulointerstitial inflammation, complex immune accumulation, and renal function in patients with lupus nephritis based on current relevant literature and the Nephroseq V5 tool (https://nephroseq.org/).

### 2.9 Statistical analysis

For continuous variables between two groups, t-test was applied if they conformed to normal distribution; Mann-Whitney U-test was used for non-normal distribution. ANOVA was applied for continuous variables between three groups. Pearson’s analysis was used to analyze the correlation between gene expression and immune cell fraction. ROC curve analysis was used to determine the diagnostic performance of the diagnostic indicators identified in the study. All statistical analyses were performed using R software (version 4.1.2) and SPSS (version 27.0) software. All statistical analyses were two-sided with P < 0.05 were regarded statistically significant.

## 3 Results

### 3.1 Identification of DEGs in LN

**Figure 1:**
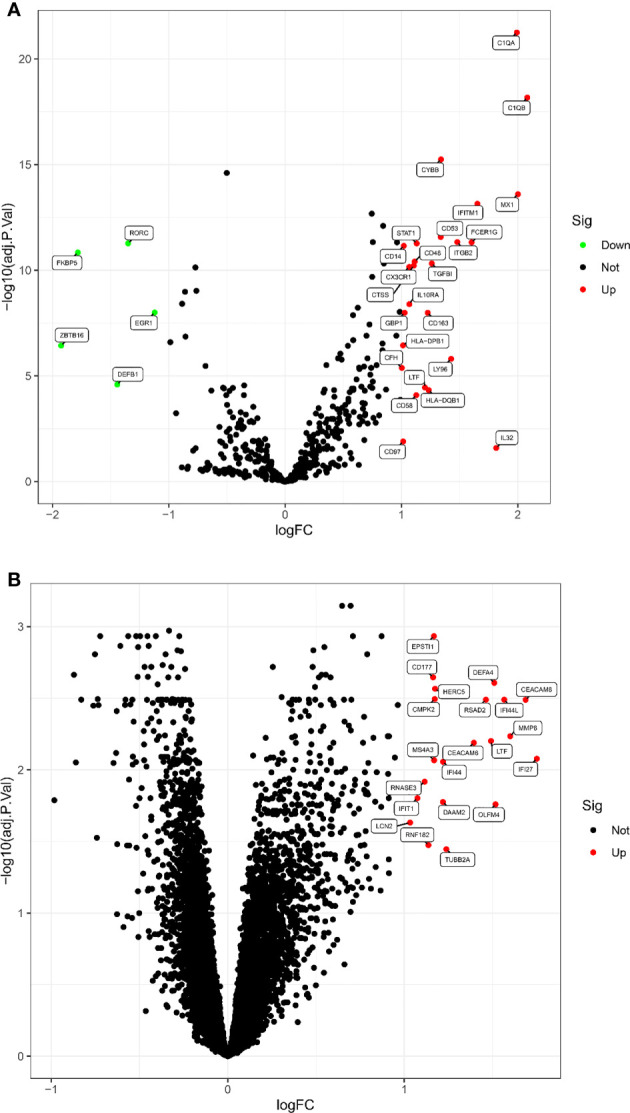
The volcano map of DEGs between LN and healthy samples. **(A)** Kidney tissue; **(B)** peripheral blood. Each dot represents a gene. Red dots indicate up-regulated gene expression; green dots indicate down-regulated gene expression; black dots indicate no significant difference between these genes in LN. The horizontal axis indicates the fold difference in gene expression in LN compared to healthy controls, and the vertical axis indicates the significance of the difference (adj. P. Value).

According to the screening criteria adjusted P value <0. 05 and |log fold change(FC)| >1, a total of 30 DEGs were screened in the LN kidney group, of which 25 were significantly up-regulated, including C1QB, MX1, C1QA, IL32, etc.; 5 were significantly down-regulated, including ZBTB16, FKBP5, DEFB1, RORC, EGR1. Volcano map of DEGs (**Figure 1A**). 21 DEGs were obtained from LN peripheral blood, all significantly up-regulated, volcano map drawn for DEGs (**Figure 1B**).

### 3.2 Functional correlation analysis

#### 3.2.1 Disease enrichment analysis of DEGs

**Figure 2:**
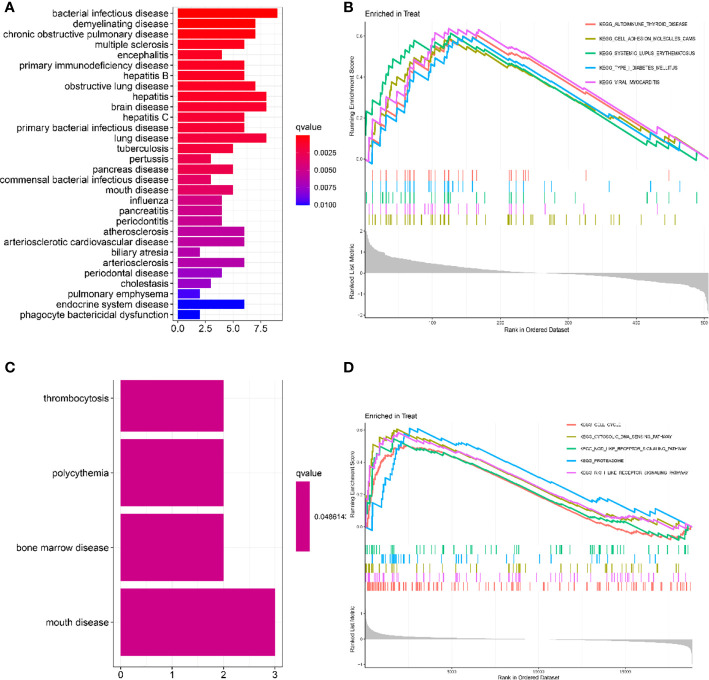
Functional enrichment analysis to identify potential biological processes by DO and GSEA. **(A, C)** Disease ontology (DO) enrichment analysis of DEGs. The vertical coordinate represents the name of the disease, the horizontal coordinate represents the number of genes enriched on the disease, and the color represents the significance of the enrichment. The redder the color, the more significant DEGs enrichment on the disease. **(B, D)** Gene set enrichment analysis (GSEA) is used to see which pathways were active in the healthy control or LN group. It consists of three main parts: The first part is the enrichment score line graph: it shows the trend of the cumulative enrichemnt score (ES) value when the analysis calculates the ES value of each gene in the gene set along the ranked list (gene list). The score at the highest peak is the ES value for that gene set. The main focus is on the peaks, with peaks at the top and ES > 0 indicating that they are active in the set; peaks at the bottom and ES < 0 indicating that they are inactive in the set. The five colors represent each of the five pathways (the top 5 in order of corrected p-value). The second part, with lines marking where members of the gene set appear in the gene sorting list, the pathways to which the different colors belong. The third part: the signal-to-noise ratio for each gene is shown as a gray frame plot. The horizontal coordinate represents the ranking in the ordered data set, and the vertical coordinate represents the Running Enrichment Score. **(A, B)** Kidney tissue; **(C, D)** Peripheral blood.

As shown in **Figure 2A**, the gene ontology disease enrichment (DO) results showed that DEGs in the LN kidney group were mainly enriched in immune class, inflammatory, renal, and cardiovascular diseases with “p values< 0.01”. Examples include primary immunodeficiency disease, vasculitis, and renal failure. DEGs in the LN peripheral blood were mainly enriched in diseases such as thrombocytosis, erythrocytosis, bone marrow diseases, and oral diseases. See **Figure 2C**.

#### 3.2.2 Significant gene enrichment pathways active in the test and control groups

In renal tissue, the top 5 significantly enriched pathways in LN compared to healthy controls were: autoimmune thyroid disease, cell adhesion molecule cam, and systemic lupus erythematosus were activated (**Figure 2B**). In peripheral blood, cell cycle, cytosolic DNA sensing pathway, NOD-like receptor signaling pathway, proteasome, and RIG-1-like receptor signaling pathway were activated in the test group compared to the control group (**Figure 2D**).

### 3.3 Diagnostic biomarker identification and validation

**Figure 3:**
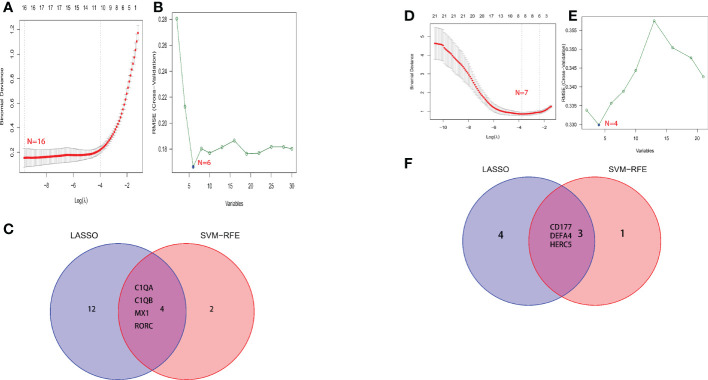
Screening process for diagnostic biomarkers of LN. **(A, D)** The LASSO model. **(B, E)** The SVM-RFE model. **(C, F)** LASSO and SVM-RFE overlap biomarkers. **(A-C)** Kidney tissue; **(D-F)** Peripheral blood.

**Figure 4:**
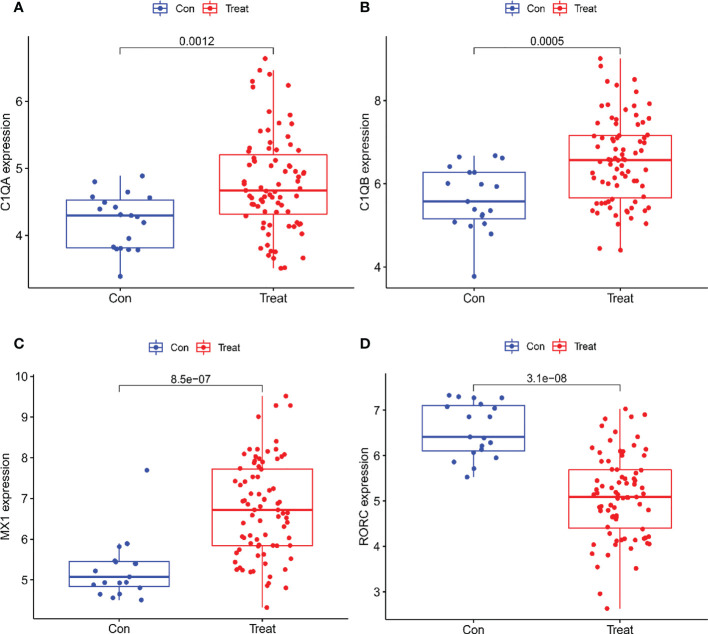
Expression of the Four biomarkers in the Validation group GSE200306 dataset. **(A)** C1QA; **(B)** C1QB; **(C)** MX1; **(D)** RORC.

**Figure 5:**
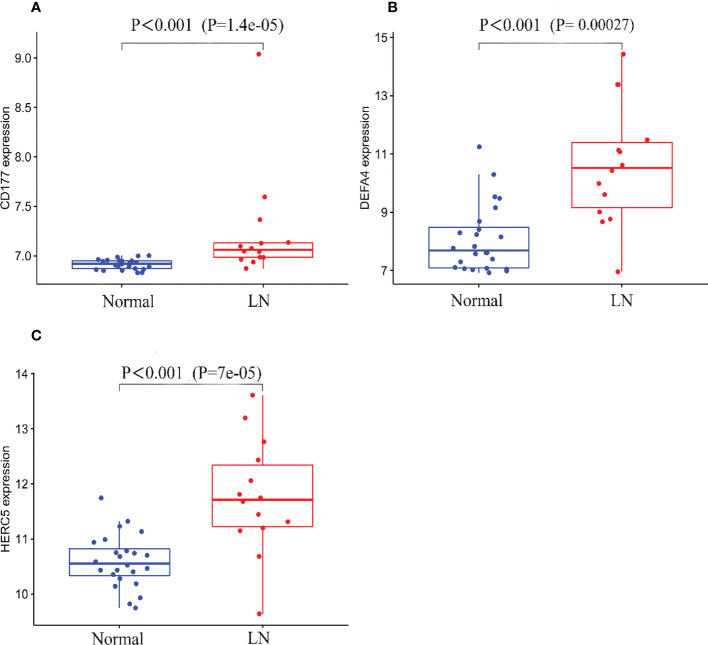
Expression of the three biomarkers in the validation group GSE81622 dataset. **(A)** CD177; **(B)** DEFA4; **(C)** HERC5.

A total of two machine learning algorithms, LASSO regression and SVM-RFE, were used to screen biomarkers in this study. In the renal group, LASSO regression identified 16 biomarkers (**Figure 3A**), SVM-RFE algorithm established 6 biomarkers (**Figure 3B**), and the overlapping genes C1QA, C1QB, MX1, RORC of both algorithms were used as diagnostic biomarkers (**Figure 3C**). To further validate the accuracy of the diagnostic biomarkers, their expression levels were further verified in the validation set GSE200306. C1QA, C1QB, MX1, and RORC were expressed at significantly higher levels in LN than in controls (p < 0.05) (**Figure 4A–D**). In the peripheral blood group, LASSO regression identified 7 biomarkers (**Figure 3D**) and SVM-RFE algorithm established 4 biomarkers (**Figure 3E**). Eventually, three overlapping genes CD177, DEFA4, and HERC5 from both algorithms were taken as diagnostic biomarkers (**Figure 3F**). To further validate the accuracy of diagnostic biomarkers, they were further validated for differences in the validation set GSE81622. All three diagnostic genes had significantly higher expression levels in LN than in controls (p < 0.05) (**Figure 5A-C**)

### 3.4 Diagnostic value of characteristic biomarkers in LN

**Figure 6:**
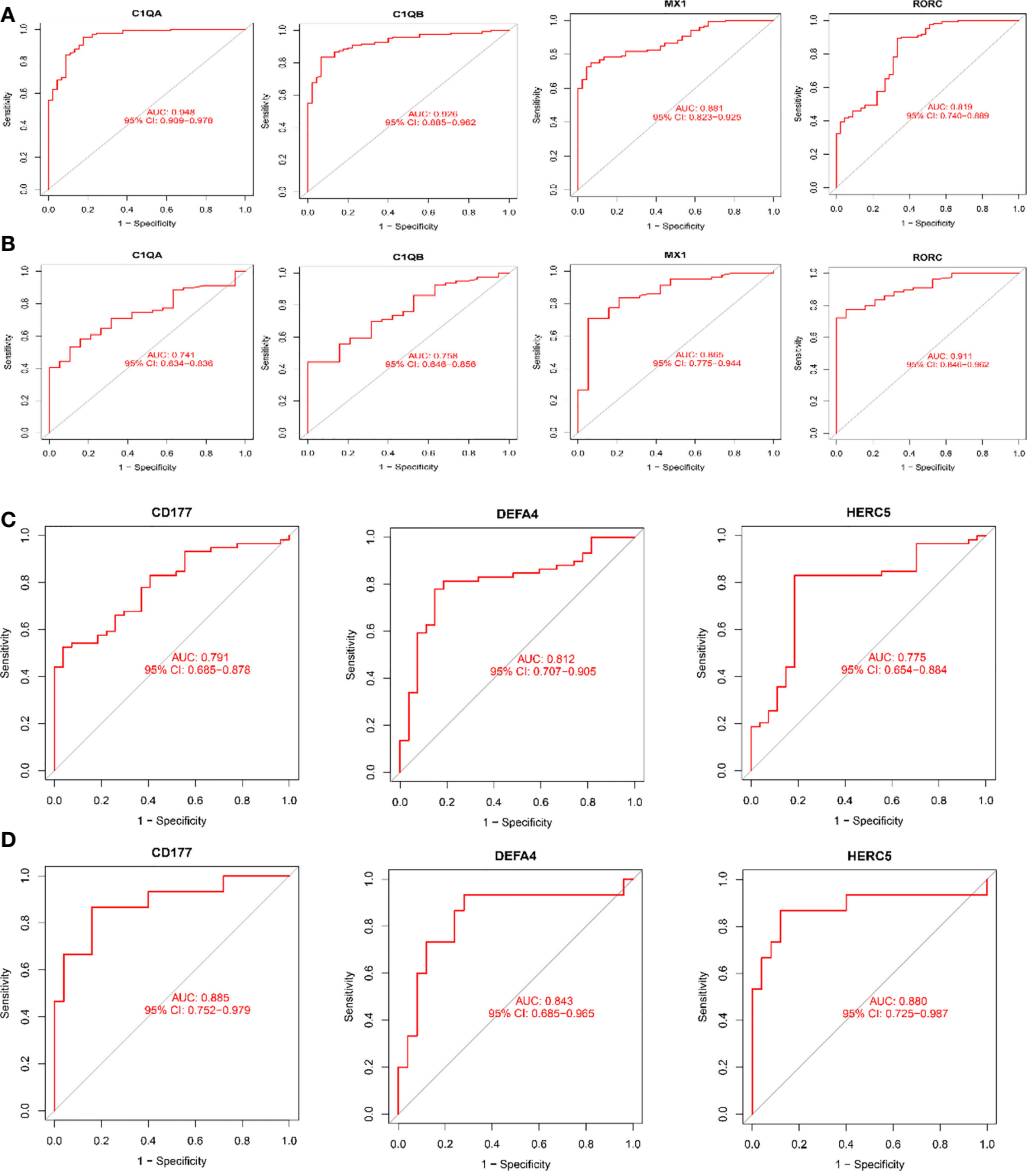
Receiver operating characteristic (ROC) curves for diagnostic validity of LN biodiagnostic markers. **(A)** ROC curves after fitting one variable for C1QA, C1QB, MX1, and RORC in the original data cohort; **(B)** ROC curves after fitting one variable for C1QB, MX1, and RORC in the GSE200306 data set. **(C)** ROC curve of CD177, DEFA4 and HERC5 after fitting one variable in the original data cohort; **(D)** ROC curve of CD177, DEFA4 and HERC5 after fitting one variable in the GSE81622 data set curve. **(A, B)** Kidney tissue; **(C, D)** Peripheral blood.

As shown in **Figure 6**, all six biomarkers screened had a high diagnostic value in LN. In the kidney tissue training group, the AUCs of the selected biomarkers were: C1QA (0.948, 95% CI: 0.909−0.978), C1QB (0.926, 95% CI: 0.885 − 0.962), MX1 (0.881, 95% CI: 0.823 − 0.925), and RORC (0.819, 95% CI: 0.740 − 0.889), as shown in **Figure 6A**; in the validation group, the AUCs of the biomarkers were C1QA(0.741, 95% CI: 0.634−0.836), C1QB(0.758, 95% CI: 0.646−0.856), MX1(0.865, 95% CI: 0.775−0.944), and RORC(0.911, 95% CI: 0.846−0.962), as shown in **Figure 6B**. The AUCs of the selected biomarkers in peripheral blood in the training group were: CD177 (0.791, 95% CI: 0.685 − 0.878), DEFA4 (0.812, 95% CI: 0.707 − 0.905), and HERC5 (0.775, 95% CI: 0.654 − 0.884), respectively (**Figure 6C**); the AUCs of the selected biomarkers in the validation group were: CD177 (0.885, 95% CI: 0.752 − 0.979), DEFA4 (0.843, 95% CI: 0.685 − 0.965), and HERC5 (0.880, 95% CI: 0.725 − 0.987), respectively (**Figure 6D**). The biomarkers showed a high diagnostic value in both the LN training and validation groups, with AUCs largely ≥0.8.

### 3.5 Analysis of immune cell infiltration

**Figure 7:**
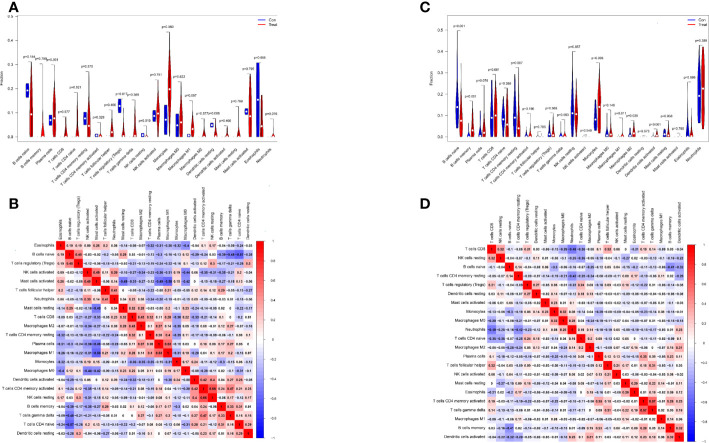
Distribution and visualization of immune cell infiltration. **(A, C)** Comparison of 22 immune cell subtypes in LN and normal tissues. **(A, C)** LN compared with 22 immune cell subtypes in normal tissue. Blue represents control group, red represents LN kidney group (**(A)** LN, **(C)** peripheral blood group). **(B, D)** Heatmap of correlations between 22 immune cell subtypes. Both horizontal and vertical axes show immune cell subtypes, and the values inside represent the correlation coefficients of immune cells. Red represents positive correlation and blue represents negative correlation. The darkest red lattice represents the highest positive correlation between the two genes, and the darkest blue lattice represents the most significant negative correlation between the two genes.

Differential analysis of immune cells in LN and normal samples showed that T cells regulatory (Tregs) (p = 0.017) were significantly lower and NK cells resting (p = 0.019) were significantly higher in LN kidney tissue than in the normal group (see **Figure 7A**). In LN blood tissues, B cells naive (p<0.001), B cells memory (p<0.001), T cells CD4 memory resting (p=0.007), Monocytes (p=0.006), Macrophages M1 (p=0.011), Macrophages M2 (p=0.039), Dendritic cells activated (p<0.001) were significantly higher than the control group (**Figure 7C**).

In addition, the correlation of 22 immune cells in all samples was analyzed (**Figure 7B, D**). There was a significant positive correlation between NK cells resting and T cells CD4 memory activated (r = 0.66) and a significant negative correlation between Mast cells activated and MacrophagesM1 (r = − 0.59) in LN kidney tissue and normal group samples (**Figure 7B**). In LN peripheral blood and normal group samples, T cells CD8 showed a significant negative correlation with Neutrophils (r=-0.49); T cells CD4 memory activated showed a significant positive correlation with T cells gamma delta (r=0.57) (**Figure 7D**).

### 3.6 Correlation of biomarkers with infiltrating immune cells

**Figure 8:**
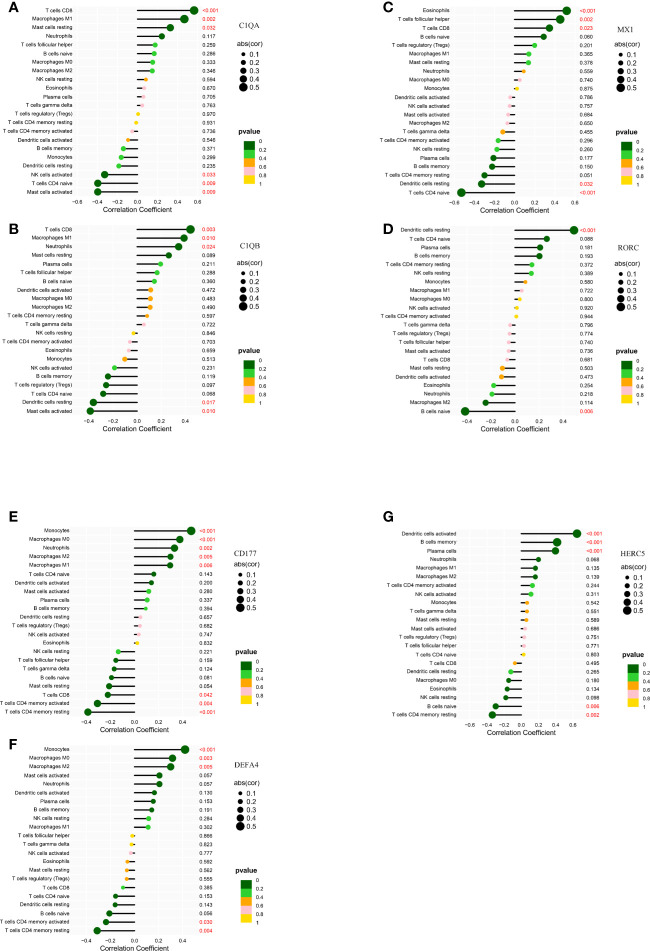
Correlation of LN kidney tissue biomarkers C1QA **(A)**, C1QB **(B)**, MX1 **(C)**, RORC **(D)** and LN peripheral blood biomarkers CD177 **(E)**, DEFA4 **(F)**, HERC5 **(G)** with infiltrating immune cells. The vertical ordinate represents the name of the immune cell and the abscissa represents the correlation coefficient. The circle size represents the absolute value size of the correlation coefficient, and the color of the circle represents the P value of the correlation test. If p value < 0.05, p values are indicated in red.

In kidney tissue, C1QA was positively correlated with T cells CD8 (R= 0.56, P= 7.94E-05)、Macrophages M1(R= 0.47, P= 0.0016) and Mast cells resting(R= 0.32, P= 0.032); and negatively correlated with Mast cells activated (R= -0.40, P= 0.0087), T cells CD4 naïve (R= -0.39, P= 0.0088) and NK cells activated(R= -0.32, P= 0.033) (**Figure 8A**). C1QB was positively correlated with MacrophagesM1 (R = 0.39, p = 0.0099), Neutrophils (R = 0.34, p= 0.024), and TcellsCD8 (R = 0.44, p = 0.0029); and negatively correlated with Dendritic cells resting (R = − 0.36, p= 0.017) and Mast cells activated (R= − 0.39, p= 0.01) (**Figure 8B**). MX1 was positively correlated with Eosinophils (R = 0.52, p = 0.00037), T cells CD8 (R = 0.35, p = 0.023), T cells follicular helper (R = 0.45, p = 0.0023); and negatively correlated with Dendritic cells resting (R = − 0.33, p = 0.032), TcellsCD4naive (R= − 0.53, p = 0.00026) (**Figure 8C**). RORC was positively correlated with Dendritic cells resting (R = 0.49, p = 0.00084); negatively correlated with B cells naive (R= − 0.42, p= 0.0056) (**Figure 8D**), and C1QB, MX1, and RORC were all correlated with Dendritic cells resting.

In peripheral blood tissues, CD177 was positively correlated with Macrophages M0 (R = 0.38, p = 0.00037), Macrophages M1 (R = 0.3, p = 0.0059), Macrophages M2 (R = 0.3, p = 0.0054), Monocytes (R = 0.47, p = 6.8e − 06), Neutrophils (R = 0.33, p = 0.002), and negatively correlated with T cells CD4 memory activated (R= − 0.31, p = 0.0042), T cells CD4 memory activated (R = − 0.39, p = 0.00026), and T cells CD8 (R = − 0.22, p = 0.042) (**Figure 8E**). DEFA4 was positively correlated with MacrophagesM0 (R = 0.32, p = 0.0032), Macrophages M2 (R = 0.3, p = 0.0051), Monocytes (R = 0.42, p = 6.1e − 05), and negatively correlated with T cells CD4 memory activated (R = − 0.24, p = 0.03), T cells CD4 memoryresting (R = − 0.31, p = 0.0039) (**Figure 8F**). HERC5 was positively correlated with B cells memory (R= 0.42, p=7.6e-05), Dendritic cells activated (R = 0.65, p = 2.2e − 11), Plasma cells (R = 0.4, p = 0.00017), and negatively correlated with B cells naive (R= − 0.3, p = 0.0056), T cell CD4 memoryresting (R = − 0.34, p = 0.0016) (**Figure 8G**). CD177, DEFA4, HERC5 were all associated with T cells CD4 memory resting.

### 3.7 Clinicopathological correlation analysis with target immune markers

To determine the clinical applicability of these markers, we consulted the Nephroseq database and related literature to obtain correlations between seven immunomarkers and clinicopathology. The summary is shown in **Table 1** and is confirmed below.

**Table 1:** Summary of biomarkers and clinicopathological features.

Biomarkers	Correlation with different pathological types of LN	Location of infiltration, tubular or glomerular	Correlation with LN patient activity	Correlation with the accumulation of immune complexes	Correlation with renal function	Correlation with traditional prognostic markers (e.g. ANA, anti-dsDNA titers, C3, C4)
C1QA	IV>III > II	Glomeruli and tubules (tubules>glomeruli)	+	++	++	+ (C3, C4)
C1QB	IV>III > II	Glomeruli and tubules (tubules>glomeruli)	+	++	++	+ (C3, C4)
MX1	ns	Glomeruli and tubules (tubules>glomeruli)	+	++	++	- (C3, C4), + (Anti-DNA antibody)
RORC	ns	Glomeruli and tubules (tubules>glomeruli)	+	- -	- -	ns
CD177	ns		+	++	++	ns
DEFA4	ns		+	++	++	ns
HERC5	ns		+	++	++	ns

(+) indicates a correlation, (++) shows a positive correlation (–), indicates a negative correlation, “ns” indicates “not yet studied”, and italic type indicate indirect evidence.

#### 3.7.1 Correlation with different pathological types of LN

**Figure 9:**
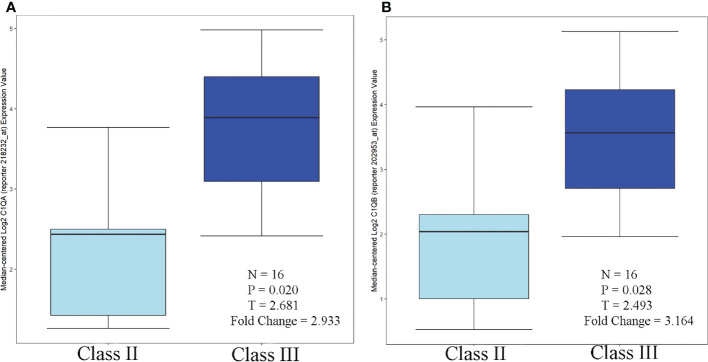
C1QA/C1QB over-expression in WHO Class III vs. WHO Class II (Lupus Nephritis Samples. **(A)** C1QA, **(B)** C1QB.

According to the Nephroseq database and related literature, seven candidate gene expressions correlated with the WHO lupus nephritis class. The expression of C1QA and C1QB was greater in class III than in class II, as shown in **Figure 9**. Compared to peripheral blood, peripheral blood C1Q antibodies have been studied more frequently, because human kidneys are more challenging to obtain. Because the expression of renal C1Q was positively correlated with peripheral blood C1Q antibodies, studies related to C1Q antibodies may have some implications. Zhu Chen et. found that in terms of quantitative C1q antibody levels, serum resistance was highest in class IV patients, followed by class III (20). Class III levels were also higher than class II but were not statistically significant. These data are also consistent with Fang et al. (21) and Cai et al. (20, 22). In summary, the expression of C1QA and C1QB was class IV > class III > class II.

#### 3.7.2 Analysis of infiltration of diagnostic genetic markers in the renal

**Figure 10:**
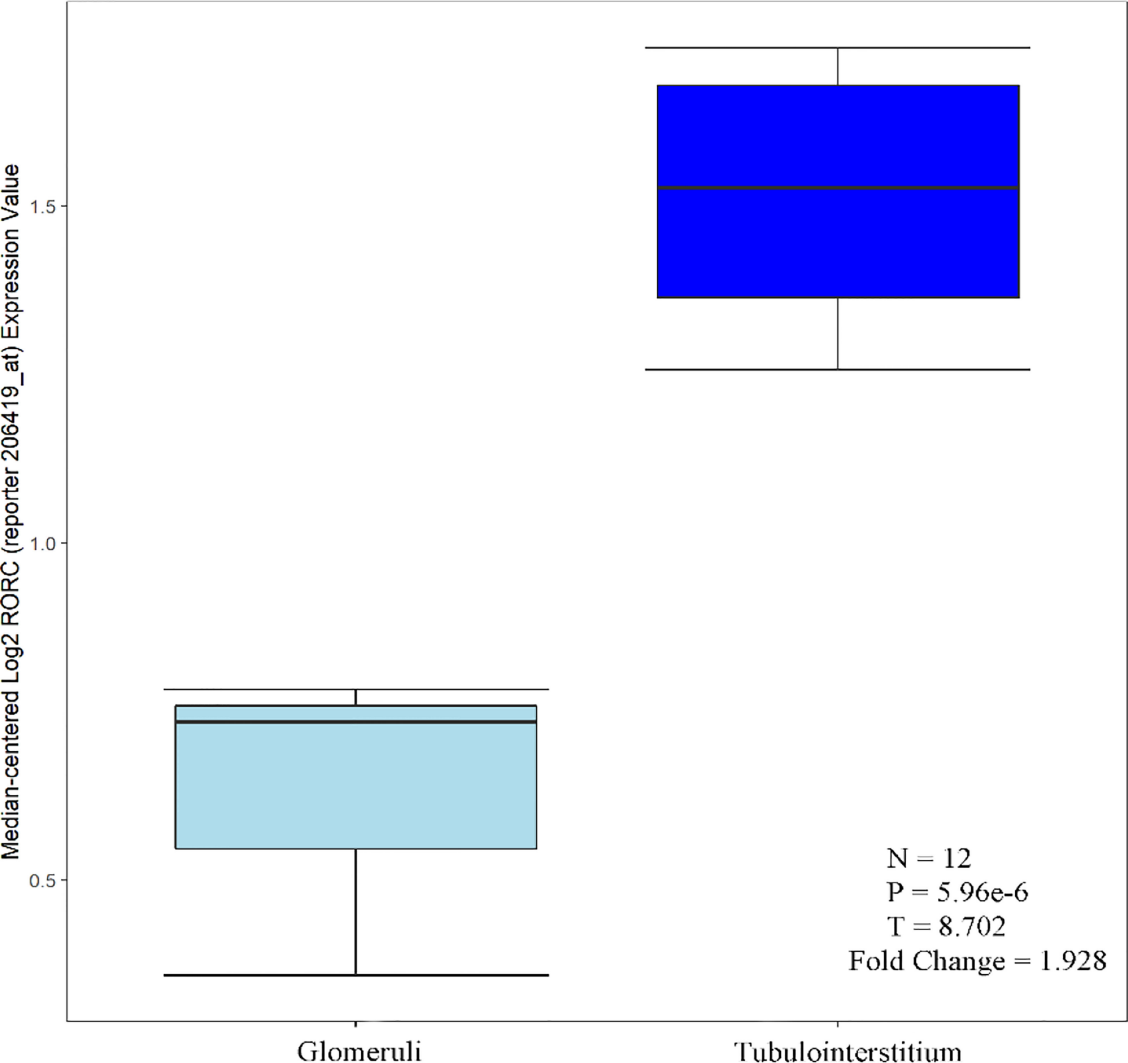
RORC over-expression in tubulointerstitium vs. glomeruli tissue.

C1QA, C1QB, MX1 and RORC were expressed in both glomeruli and tubules, with greater tubular than glomerular expression. Based on available literature and the Nephroseq database. Tao Liu et al. performed immunohistochemical analysis of kidneys from LN patients and healthy controls, and C1q was expressed in both glomeruli and tubules. Moreover, C1q protein levels were higher in the renal tubules (23), consistent with Desiree Tampe et al. Desiree Tampe observed a significant induction of glomerular, tubulointerstitial compartments, C1QA, and C1QB expression levels in patients with LN compared to healthy controls (24). MX1 is one of the IFN-I-inducible genes. Increased expression of the MX1 gene was detected in renal cells, PBMC, and renal tubules of patients with LN (25–28). Based on the Nephroseq database, it is known that RORC was distributed in both glomeruli and tubules, and the expression in tubules was greater than that in glomeruli (**Figure 10**).

#### 3.7.3 Correlation with the activity and recurrence frequency of LN

Based on the current study, the relevant literature directly or indirectly confirms that the expression of C1QA/B (29–33), MX1 (25, 27), RORC (34), CD177 (35, 36), DEFA4 (37, 38), and HERC5 (39, 40) is strongly correlated with SLE/LN activity in a broad sense. However, there are a few studies with different results. In terms of LN activity-related and relapse rates, N Marto et al. found a higher prevalence of anti-C1q antibodies in patients with active LN than in those without the renal disease (74% V 32%, p<0.0001) (41). GabriellaMoroniMD et al. showed that the onset or recurrence of LN was associated with elevated anti-C1q antibody titers in the previous six months (42). High levels of C1q antibodies appear two to six months before the onset of LN and decrease or become undetectable after successful treatment (41, 43–49). In addition, RORC also showed significant downregulation in patients with LN in remission (50). In summary, C1QA, C1QB, MX1, RORC, CD177, DEFA4, and HERC5 can somewhat predict the prognosis of LN (37, 38, 40, 41, 51–53).

#### 3.7.4 Relationship with immune complexes

LN begins with the *in situ* formation and deposition of immune complexes. Subsequently, immune complexes deposition triggers a complex series of events involving *in situ* activation of complement (C1q A, C1QB), Fc receptors, and adhesion molecules, leading to the recruitment and activation of inflammatory cells and ultimately kidney injury (54, 55). Therefore, we hypothesized that the gene expression of seven candidate genes, except RORC, is positively correlated with immune complexes. The deposition of immune complexes activates their expression. The available literature confirms the following.

① C1q is crucial in clearing immune complexes and apoptotic cells (56–58). This clearance may be impaired by low C1q levels of apoptotic cells and cellular debris, which are sources of autoantigens (59, 60). The immune response induced by the inefficient clearance of apoptotic cells and cellular debris gives rise to autoantibodies.
② MX1 and HERC5 are interferon-inducible genes positively associated with immune complexes. The immune complexes are endogenous IFN inducers (61). Precipitates of immune complexes in the kidney activate a series of inflammatory responses and the production of type I interferon (62–64). Type I interferon binds to the type I interferon receptor (IFNαR), which induces interferon-stimulated genes (ISG) (including MX1, HERC5) (65).
③ The relationship between RORC, CD177, DEFA4 and immune complexes has not been reported for the time being.

#### 3.7.5 Relationship with renal function

**Figure 11:**
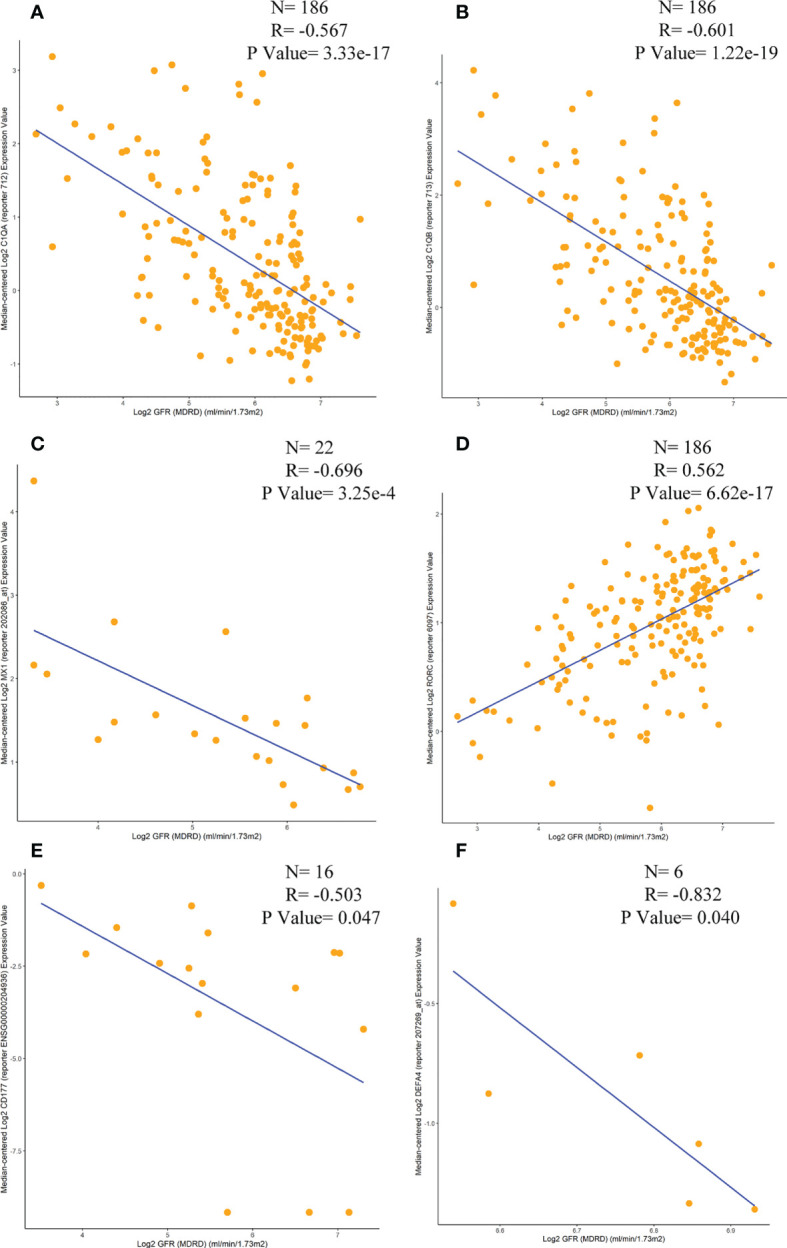
Correlation analysis of the expression levels of C1QA, C1QB, MX1, RORC, CD177 and DEFA4 with GFR. **(A)** C1QA, **(B)** C1QB, **(C)** MX1, **(D)** RORC, **(E)** CD177, **(F)** DEFA4.

Correlations between six immunomarkers and renal function (glomerular filtration rate, GFR) were obtained from the Nephroseq database. Scatter plots showed that all were negatively correlated with GFR except for RORC (**Figure 11**), consistent with relevant contemporary research by Zhu Chen et al. (20). Anti-C1q was positively correlated with renal activity indices, including intra-capillary hypercellularity, glomerular leukocyte infiltration, and nuclear rupture/fibrin-like necrosis. It was negatively correlated with chronic indices, such as glomerulosclerosis and interstitial fibrosis.

#### 3.7.6 Correlation with traditional prognostic markers (e.g., ANA, anti-dsDNA titers, C3, and C4)

There are few articles on the association of peripheral blood genetic markers CD177, DEFA4, and HERC5 with traditional prognostic markers (e.g., ANA, anti-dsDNA titers, C3, C4), although there are relevant theoretical underpinnings. Future studies could be conducted in this direction to clarify the correlation between the two further. The C1Q antibody is currently relatively well-studied. Rosalie M. Sterner et al. found that the bound C1 complex could cleave C4 and C2 to form the C3 convertase complex, which could cleave C3 into C3a and C3b, with C3b contributing to phagocytosis of regulatory substances and clearance of immune complexes (4, 66). Tao Liu et al. further found that the levels of C1q and C4 were positively correlated with the levels of C3 in the blood (41). In addition, N Marto et al. found that peripheral blood anti-C1q titers were negatively correlated with C3 and C4 levels and positively correlated with anti-dsDNA (22, 41, 67). C1Q in serum was negatively correlated with anti-C1Q antibodies. In summary, C1q expression was positively correlated with the levels of C3 and C4. MX1 was negatively correlated with C3 and C4 and positively correlated with Anti-DNA antibodies (25).

#### 3.7.7 Applications

The applications of the genetic biomarkers screened in this study are broadly divided into two major blocks: one for diagnosing LN, assessing disease status, and predicting the prognosis of LN; and the other for guiding clinical drug use and precise treatment.

##### 3.7.7.1 Diagnosis and assessment of the activity

① Anti-C1q antibodies can be used to predict the onset of nephritis (21, 41, 68–70). A previous meta-analysis showed that anti-C1q antibodies had good sensitivity and specificity in the diagnosis of LN (0.58 and 0.75, respectively) (71). In addition, high titers of anti-C1q autoantibodies were associated with active LN with a sensitivity of 97% and a specificity of 92% (41, 42, 68, 69, 72). On the other hand, the C1q gene can be used for early screening. A quantitative or functional defect in C1q in pure congeners has been reported to be a strong disease susceptibility factor, with more than 90% of patients presenting with SLE and SLE-like symptoms (57, 73–75).

② Upregulation of ISG in peripheral blood is a hallmark of SLE and has been associated with SLE disease activity, playing an important role in the pathogenesis of SLE (27, 38, 51, 76, 77). MX1 is of great importance as a biomarker for SLE (78).

③ CD177 and DEFA4 are neutrophil subsets that correlate with the onset, severity and prognosis of SLE/LN (79–81). In renal biopsies, neutrophils were found to be a marker of glomerular activity in SLE, while detection of neutrophil-derived proteins in urine is an alternative marker of disease activity (35). An increase in circulating LDG population was also observed in adolescent SLE patients, which correlated with dsDNA antibody concentrations and disease activity scores. This suggests that the neutrophil subpopulation (CD177, DEFA4) may be a useful biomarker (36).

④ Kennedy et al. reported that the HERC5 gene could be used as an IFN biomarker in SLE patients (82). A recent study also concluded that IFN characteristics may be a better biomarker of overall disease severity than disease activity, and that IFN-High patients tend to have a more severe disease course (51, 83).

##### 3.7.7.2 Treatment

① C1Q: SLE patients with C1q deficiency were successfully treated by fresh frozen plasma, which transiently restored complement activity and reduced circulating immune complexes (84–86).

② MX1: Clinically, a number of biologic agents targeting type I interferon have emerged (87). In addition, Mx1 gene expression in peripheral blood cells has been used as a sensitive biomarker for LN treatment, identifying patients who have responded well to anti-I interferon therapy (27, 88).

③ RORC: RORγt is a core transcription factor for Th17 cell differentiation. Many studies suggest that RORγt may be a potential therapeutic target for Th17-mediated autoimmune diseases (89–93). Currently, several pharmaceutical companies are developing RORgt blockers and more than 200 small molecule compounds have been patented. They can inhibit RORγt activity, block Th17 differentiation and improve autoimmune disease manifestations (90, 94). Several Phase I and Phase II studies are already underway (95, 96).

④ CD177, DEF4: NETosis intervention improves the performance of experimental SLE (97, 98). Neutropenia has been reported in 20-40% of SLE patients and SLE patients treated with immunosuppressive drugs may also be at risk of developing neutropenia (99, 100). Breaking this pathway may lead to the development of new drugs. Anti-CD20 therapy has been shown to improve SLE in humans (101).

⑤ HERC5: Several drugs targeting the type I interferon pathway in SLE and LN are currently being investigated. Several drugs targeting the type I interferon pathway in SLE and LN are being investigated, including anti-interferon monoclonal antibodies (sifalimumab, rontaziumab), and monoclonal antibodies targeting the type I interferon receptor (anifolumab) (102–104). Also, many clinical trials have included the measurement of IFN characteristics in their studies. Because the underlying disease mechanisms may differ between SLE patients with IFN-High and IFN-Neg, interferon scores have been applied to stratify patients for treatment.

## 4 Discussion

Due to the invasive nature of renal biopsy and the non-specific nature of LN treatment methods, LN cannot be treated precisely, and there is a steady trend of LN patients progressing to ESRD worldwide (105). How to identify LN early, intervene in time, and reduce LN patients into chronic renal failure is significant. The traditional biomarkers of LN, C3, C4, ds-DNA and anti-Sm, have some significance in the evaluation of LN disease, but they all have some limitations (106–110). Therefore, it is urgent to develop new biomarkers to predict the renal pathology of LN patients. The pathogenesis of LN is not clear in modern medicine, but it is generally believed that renal infiltrating immune cells play an important role in the pathogenesis of LN (111–113). Significant accumulation of various types of immune cells, including T cells, B cells, plasma cells, NK cells, and macrophages, has been observed in the kidneys of active LN and lupus-prone mice (114–116). Epidemiological studies have shown significant familial aggregation of LN, suggesting that genetic factors play an important role in the development of LN and disease progression (117). Up to now, a large number of related genes that may be involved in the pathogenesis of LN have been reported, but few studies have focused on abnormally expressed gene biomarkers associated with immune infiltration between LN and normal tissues. Therefore, we aimed to identify candidate diagnostic biomarkers for LN and investigate the role of immune cell infiltration in LN.

### 4.1 Correlation of DEGs with LN

In this study, DEGs in kidney tissue and peripheral blood of LN were analyzed by combined GEO database multi-chip. A total of 30 DGEs were screened in kidney tissues, 25 of which were upregulated, mainly CD163, CFH, CX3CR1, HLA-DPB1, HLA-DQB1, IFITM1, IL10RA, etc.; 5 were downregulated, mainly RORC, etc. A total of 21 DGEs were screened in LN peripheral blood, all of which were significantly upregulated. The main ones were IFI44L, LCN2, CD177, etc. DEGs were all closely related to LN.

Professor Xueqing Yu of Sun Yat-sen University found that several independent susceptibility loci such as HLA-DPB1 and HLA- DQB1 are closely associated with the development of LN (118). Also, HLA- DQB1 is a susceptibility gene for SLE (119). Differentiated antigen cluster 163 (CD163) is expressed in monocytes/macrophages, a marker of macrophage activation, and has the ability to mediate the clearance of free hemoglobin by macrophages, thereby reducing kidney damage from the reaction of heme iron with endogenous hydrogen peroxide to generate free radicals (120, 121). It has the ability to modulate immunity, scavenge tumor necrosis factor-like weak apoptosis-inducing factors, activate inflammatory responses, and induce cell proliferation and apoptosis (122, 123). Several studies have shown that CD163+ macrophages infiltrate significantly in patients with LN (124, 125), and the number of CD163+ macrophage infiltration is closely related to the activity of LN (125). Studies on other macrophages are relatively scarce and need to be further explored in the future. CX3CR1 is widely expressed in the renal tissue of patients with LN and can be used as one of the alternatives to renal biopsy (126). CX3CR1 is involved in the pathogenesis of human type IV LN (127). Administration of an analogue of fractalkine as an antagonist in MRL/lpr lupus mice significantly inhibited the development and progression of their LN (128). An investigation by Professor Wang Fengmei of Peking University showed that serum CFH levels were closely related to LN disease activity and that CFH deficiency accelerated the development of LN (129). CFH-related genes are also associated with SLE susceptibility (130). IFN is a major pathogenic factor in systemic lupus erythematosus (SLE) and LN (131). More than 20 cross-sectional case-control studies have shown that the expressions of IFI27, IFI44, IFI44L, IFIT1, PRKR and RSAD2 are proportionally clustered in LN (131). IFI44L promoter methylation can be used as a blood biomarker for LN (132–134). The protein encoded by IL10RA is a receptor for interleukin 10 (IL-10) (135). Overexpression of IL-10 in LN renal tissue and peripheral blood is highly correlated with the onset and activity of LN (136, 137). FKBP5 and EGR1 are closely associated with SLE, but the direct correlation with LN needs to be further confirmed (138, 139).

The interferon-inducible transmembrane protein (IFITM) gene family includes IFITM1, which plays a role in a number of biological activities such as interferon-homotypic cell adhesion function and anti-proliferative activity of cells (140–143). Lipocalin-2 (LCN2) is an indicator of the severity of LN and plays a key role in the immune response, which promotes Th1 cell differentiation exacerbating LN in an autocrine or paracrine manner, mainly through the IL-12/STAT4 pathway (144). Urinary LCN2 can be used as an early biomarker of LN (145, 146). RSAD2 is an interferon-inducible iron-sulfur cluster-binding antiviral protein. In a study of genome-wide DNA methylation in LN, methylation of the CpG site of the RSAD2 gene was shown to be highly correlated with LN (147). TGFB1 and MMP8 may be involved in LN through a mediated matrix pathway (148). The relationship between diagnostic biomarker genes and LN is detailed in the biomarkers section.

### 4.2 Functional correlation analysis

The disease enrichment analysis of LN kidney tissue DGEs was mainly enriched in immune class, inflammatory, renal and cardiovascular diseases, such as: primary immunodeficiency disease, vasculitis, renal failure, renal cancer, and renal disease. This fits in previous studies. In terms of the pathogenesis of SLE, multiple factors such as multiple genes and environmental factors are involved (149), and viral infections are often used as environmental triggers of SLE (150). In other words, LN is essentially an immune-mediated inflammatory response. In terms of the symptoms and outcomes of LN, there is a significant correlation between LN and cardiovascular disease (151), and 20.8% of deaths in LN patients are caused by cardiovascular disease (152). Many LN patients end up with chronic kidney disease (CKD) and ESRD despite receiving potent anti-inflammatory and immunosuppressive therapy.

Differential genetic disease enrichment in peripheral blood is mainly associated with hematologic disorders, bone marrow. is mainly associated with hematologic disorders, bone marrow. For example, erythrocytosis, thrombocytosis, bone marrow disorders, oral diseases, etc. SLE often involves the blood system, and hematological abnormalities are a common manifestation of SLE (153). Hypercoagulation is often accompanied in LN, which may be related to its immune complexes which can promote platelet aggregation, form thrombus, and cause microcirculation disorders. One of the more controversial points is that thrombocytosis may be an indicator of disease activity and responsiveness. Because about a quarter of SLE patients usually show thrombocytopenia, thrombocytosis is an unusual finding. LN can also be accompanied by oral ulcers. For example, a retrospective study conducted by a tertiary medical center in Assam, India, showed that 176 patients with LN had a 31.8% probability of having a concomitant oral ulcer (154). SLE, especially patients with LN, is often accompanied by osteoporosis, which may be related to the long-term use of prednisone (155). Ramsey et al., 1994, in the US National Health Survey, found that the incidence of fractures in patients with SLE was five times higher than in the normal population (156).

GSEA results indicate that the immune response plays a crucial role in LN. GSEA enrichment of kidney tissue mainly involved immunity with inflammation. For example, the five pathways of autoimmune thyroid disease, cell adhesion molecules cams, systemic lupus erythematosus, type 1 diabetes mellitus, and viral myocarditis were activated. Cell adhesion molecules (CAM) play a key role in inflammation, immune response, and thrombosis (157). Intercellular adhesion molecule-1 (ICAM-1) and vascular cell adhesion molecule-1 (VCAM-l) are two CAMs that are currently receiving the most attention. In the autoimmune MRL/lpr model in LN mice, it was found that the severity of renal histopathology was correlated with the expression of VCAM-1, and ICAM-1 was also used as a major marker of renal inflammatory activity (158). Blocking this pathway may inhibit the pathological infiltration of LN inflammatory cells (159). In clinical trials, the expression of ICAM-1 and VCAM-1 in glomerular endothelial cells and cells, serum and urine of LN patients was significantly increased, which was positively correlated with LN activity and played an important role in the development of LN (160–162). At present, anti-adhesion molecule antibodies have achieved a certain effect in rheumatism (163), vascular remodeling, heart and cerebral ischemia-reperfusion (164, 165) and other aspects, and the field of nephropathy urgently needs to be developed. The results of LN peripheral blood GSEA enrichment analysis showed that the pathways were mainly involved in immune, cell cycle. For example, cell cycle, cytosolic DNA sensing pathway, NOD-like receptor signaling pathway, proteasome, and RIG-1-like receptor signaling pathway were activated. Inflammatory response or cellular injury triggers abnormal proliferation of LN tethered cells, activates the cell cycle and ultimately leads to glomerulosclerosis and end-stage renal failure. Interference with the cell cycle can prevent cell proliferation or promote apoptosis (166). For example, tacrolimus and motilmic acidophilus, commonly used drugs in LN, inhibit the proliferation of thylakoid cells by acting on different phases of the cell cycle of thylakoid cells and thus slow down the progression of glomerular disease (167, 168). The transmission mechanism of cytoplasmic DNA is the molecular basis for the immune system to produce an immune response. Aberrant self-DNA accumulation and cellular type I interferon (IFN-I) secretion are the two main parts in the pathogenesis of LN (169, 170), while cellular sensors play an important role in the process of extracellular DNA triggering IFN-I (171). Various DNA sensors have been reported so far, but specific cytoplasmic DNA sensors involved in LN’s own DNA-induced autoimmune response are yet to be further investigated. Nucleotide-binding oligomerization domain NLR and RIG-1 like receptors are involved in the pathogenesis of SLE/LN, which mainly stimulate cytokine production by activating innate immunity through the uptake of immune complexes (ICs) between antinuclear antibodies and nucleic acids into cells (172, 173). In addition, the NLR family also drives key molecules of the inflammatory response through the formation of “inflammasomes”, resulting in transcriptional induction of type 1 IFNs and other inflammatory cytokines (150). This information is essential to improve therapeutic strategies for LN, such as the production of drugs that target interferon or interferon receptors. Proteasome inhibition is a new therapy for LN and plays an important role in the treatment of LN, such as cyclophosphamide, rituximab, and bortezomib (174).

### 4.3 Biodiagnostic markers screened by machine learning

Based on two machine learning algorithms, LASSO and SVM-RFE, four biomarkers with diagnostic value were screened in kidney tissues: C1QA, C1QB, MX1, and RORC. C1q (C1qA, C1qB and C1qC) is the first component of the classical pathway of complement activation and plays an essential role in SLE. In recent years, domestic and international studies have shown a significant correlation between C1q deficiency and abnormal C1qAb and the onset, disease activity and pathological type transformation of SLE, especially LN (42, 175, 176). Genetic defects in C1q are susceptibility factors for SLE, and close association of LN with C1q deficiency has been demonstrated in both human SLE and C1q-deficient animal models (4, 175, 175, 177–179). Based on a systematic review of reported cases, approximately 75-93% of patients with C1q deficiency have SLE or SLE-like disorders (74, 180). In 1998, in a review of patients with C1q deficiency, Walport et al. found that 38 of 41 subjects (93%) exhibited a clinical syndrome closely related to SLE (57). In 2011, Schejbel et al. found that 88% of the 63 patients reported with C1q deficiency had SLE/SLE-like disease (181). In 2014, Mihaela Stegert et al. reviewed the overall prevalence of SLE/SLE-like syndrome in all 71 patients with C1q deficiency reported as 77.5% (182). In the C1q-deficient SLE mouse model (MRL/Mp-+/+), C1q deficiency in mice can also lead to autoimmune diseases such as SLE (177). On the one hand, it is due to the impaired phagocytic clearance of apoptotic cells, and on the other hand, C1q deficiency significantly enhances the production of anti-C1q antibodies, leading to the development of proliferative immune complex glomerulonephritis (183, 184). It explains the lack of association of C1q with SLE/LN (177). The association between C1QA and C1QB in the renal biomarker screen may further confirm the importance of the complement cascade in LNs. According to related studies complement cascade activation in SLE patients leads to hypocomplementemia and deposition of complement components at sites of tissue damage (4, 184). Human mucovirus resistance protein 1 (Mx1), one of the type I interferon (IFN)-inducible genes (27), is a type I IFN-dependent transcript that plays a major role in apoptosis and cytokine-mediated cell signaling. In LN, MX1 is used as a potential marker for diagnosing peripheral blood LN activity (25, 28) and is also considered as a susceptibility gene for SLE (25, 28). RORC is a key factor in coordinating the transcription of genes encoding IL17 and plays a key role in the regulation of inflammatory response (185). Studies have confirmed that it plays an important role in the dysregulated immune response associated with SLE (50).

Three biomarkers with diagnostic value were screened in peripheral blood: CD177, DEFA4, HERC5. CD177 is an important neutrophil gene encoding neutrophil membrane glycoprotein, but its function in neutrophils is not fully understood. Neutrophils play an active role in driving autoimmune response and tissue damage in SLE. We speculate that CD177 is mainly involved in the pathogenesis of SLE/LN by regulating NET and/or neutrophils (186). Current studies of CD177 are mainly focused on anti-neutrophil cytoplasmic antibody (ANCA)-related systemic vasculitis (187). In the future, we should pay attention to related research to fill the gap in LN. DEFA4 is expressed at a higher level in patients with active SLE (188), and is involved in immune-mediated tissue damage caused by SLE autoantibody deposition (189). In addition, DEFA4 is related to early granulocyte production and plays a role in the maturation of neutrophils and the formation of neutrophil extracellular traps (NETs). In other words, DEFA4 may be involved in the pathogenesis of LN by affecting neutrophils (37). HERC5 is an interferon-inducible gene (190–193). CoitP et al. found that HERC5 is hypomethylated in lupus patients with renal involvement (194), and Lingling Shen et al. further validated the crucial role of HERC5 in the pathogenesis of LN by PCR (qRT-PCR) and enzyme-linked immunosorbent assay (ELISA) (134).

### 4.4 Types of immune cell infiltration

Analysis of LN samples and normal samples using CIBERSORT revealed that a variety of immune cell subtypes were closely related to important biological processes in LN. In kidney tissue, T cells regulatory (Tregs) infiltration was reduced; NK cells resting infiltration was increased; in blood samples, B cells memory, T cells CD4 memory resting, Monocytes, Macrophages M1 and Macrophages M2 Dendritic cells activated infiltration was increased; B cells naive infiltration was decreased. The screened biomarkers were correlated with infiltrating immune cells, and the biomarkers C1QB, MX1, and RORC were correlated with Dendritic cells resting in kidney tissues. Peripheral blood tissue CD177, DEFA4, HERC5 were correlated with T cells CD4 memory resting. The specific mechanism can be traced back to the fact that LN is often triggered by abnormal immune system, involving a variety of immune cells, cytokines and related pathways (195). T cells e.g., CD4^+^, CD8^+^) play a central role in the pathogenesis of LN (196). In animal experiments, CD4 was shown to induce LN when infused into lupus-prone mice with CD4^+^ T cell lines (197). In the clinic, Masutani et al. found that CD4+ cells were the predominant cell type in grade I, IV and V renal infiltrates in patients with lupus nephritis (198, 199). CD4^+^ T is involved in LN pathogenesis and has been demonstrated to mediate LN inflammation (200), which exerts its function mainly by secreting cytokines (e.g., IFN-γ, IL-17 and IL-10, etc.) upon antigen activation and by transmitting tissue inflammation (201). In addition, it was reported that CD8+ T lymphocyte infiltration was predominant around the glomeruli of patients with LN, and the infiltrating interstitial cells were mainly CD4+ve T cells or CD8+ve (199, 202–205). Nataly Manjarrez-Orduño et al. found that a subpopulation of LN exhibited enhanced CD8+ terminal differentiation, suggesting a pathological role for CD8+ T cells in a specific subpopulation of LN and laying the groundwork for CD8-targeted therapy (206).

At the same time, relevant studies have shown that RNA sensing by traditional dendritic cells (DC) is central to LN development (207). Dendritic cells are the most potent antigen presenting cells known so far and are essential in the induction and maintenance of immune tolerance (208). DC have two subpopulations of myeloid dendritic cells (mDCs) and plasmacytoid dendritic cells (pDCs) (209). Regardless of which subpopulation is dysregulated, it will lead to tolerance disruption and self-attack, resulting in the development of LN (210). Both peripheral DC subpopulations are expected to be alternative diagnostic tools for invasive biopsy. Current SLE therapies, both conventional and biological, target specific subsets of adaptive immune cells, primarily B cells, T cells, and B and T cells are still considered to be the culprits of immune dysregulation in SLE (211), but DCs are paramount in establishing and maintaining peripheral tolerance (212, 213). DCs have a superior ability to acquire and process antigen for delivery to T cells and express high levels of co-stimulatory or co-inhibitory molecules that determine immune activation or non-response. This further highlights the importance of developing DCs in LN therapy, such as the administration of DC vaccines that may bring new hope for the treatment of LN (214).

Although this study used a large GEO dataset of LN kidney tissue and peripheral blood to explore immune cells and immune-related genes in LN and identified six diagnostic gene biomarkers with diagnostic and predictive value in patients with LN, there are still some limitations to be addressed. First, the study was retrospective and did not allow for first-hand, clinically important information. Second, the CIBERSORT algorithm has limitations in distinguishing Th17 from other CD4+ T cell subpopulations and cannot further analyze the correlation between the CD4+ T subpopulation Th17 and LN biomarkers. Third, bioinformatic determination of LN biomarkers and the function of immune cell infiltration requires further confirmation in a larger sample size prospectively.

## 5 Conclusion

C1QA, C1QB, MX1 and RORC (kidney); CD177, DEFA4 and HERC5 (peripheral blood) can be used as new candidate molecular markers for LN. Dendritic cells resting and T cells CD4 memory resting may be involved in LN development. These immune cells have the potential to be new targets for immunotherapy in LN patients.

## Data availability statement

The datasets presented in this study can be found in online repositories. The names of the repository/repositories and accession number(s) can be found in the article/**Supplementary Material**.

## Author contributions

HTY, KY directed the research and revised the manuscript; LW, ZY, HXY performed the research and wrote the paper; WL modified the tables and figures and revised the manuscript; RW was involved in finding and organizing the literature during the rework. All authors contributed to the article and approved the submitted version.

## Funding

This work is supported by the National Natural Science Foundation of China (81873263).

## Acknowledgments

The authors are thankful to Tianjin University of Traditional Chinese Medicine for the help in conducting this study.

## Conflict of interest

The authors declare that the research was conducted in the absence of any commercial or financial relationships that could be construed as a potential conflict of interest.

## Publisher’s note

All claims expressed in this article are solely those of the authors and do not necessarily represent those of their affiliated organizations, or those of the publisher, the editors and the reviewers. Any product that may be evaluated in this article, or claim that may be made by its manufacturer, is not guaranteed or endorsed by the publisher.

## Abbreviations

ESRD, End-stage renal disease; LN, Lupus nephritis; DEGs, differential express genes; LASSO, least absolute shrinkage and selection operator; SVM-RFE, support vector machine-recursive feature elimination; AUC, area under curve; DO, gene ontology disease enrichment; GSEA, gene set enrichment analysis; NLR, NOD-like receptor.
